# Alone at last! – Heterologous expression of a single gene is sufficient for establishing the five-step Weimberg pathway in *Corynebacterium glutamicum*

**DOI:** 10.1016/j.mec.2019.e00090

**Published:** 2019-04-10

**Authors:** Christian Brüsseler, Anja Späth, Sascha Sokolowsky, Jan Marienhagen

**Affiliations:** Institute of Bio- and Geosciences, IBG-1: Biotechnology, Forschungszentrum Jülich GmbH, Jülich, D-52425, Germany

**Keywords:** *Corynebacterium glutamicum*, D-xylose, Weimberg pathway, α-ketoglutarate

## Abstract

*Corynebacterium glutamicum* can grow on d-xylose as sole carbon and energy source via the five-step Weimberg pathway when the pentacistronic *xylXABCD* operon from *Caulobacter crescentus* is heterologously expressed. More recently, it could be demonstrated that the *C. glutamicum* wild type accumulates the Weimberg pathway intermediate d-xylonate when cultivated in the presence of d-xylose. Reason for this is the activity of the endogenous dehydrogenase IolG, which can also oxidize d-xylose. This raised the question whether additional endogenous enzymes in *C. glutamicum* contribute to the catabolization of d-xylose via the Weimberg pathway. In this study, analysis of the *C. glutamicum* genome in combination with systematic reduction of the heterologous *xylXABCD* operon revealed that the hitherto unknown and endogenous dehydrogenase KsaD (Cg0535) can also oxidize α-ketoglutarate semialdehyde to the tricarboxylic acid cycle intermediate α-ketoglutarate, the final enzymatic step of the Weimberg pathway. Furthermore, heterologous expression of either *xylX* or *xylD*, encoding for the two dehydratases of the Weimberg pathway in *C. crescentus*, is sufficient for enabling *C. glutamicum* to grow on d-xylose as sole carbon and energy source. Finally, several variants for the carbon-efficient microbial production of α-ketoglutarate from d-xylose were constructed. In comparison to cultivation solely on d-glucose, the best strain accumulated up to 1.5-fold more α-ketoglutarate in d-xylose/d-glucose mixtures.

## Highlights

• *C. glutamicum* requires only one additional dehydratase to grow on d-xylose.
• XylX or XylD can be used to establish the Weimberg pathway in *C. glutamicum*.
• cg0535 (*ksaD*) encodes for an α-ketoglutarate semialdehyde dehydrogenase.
• *C. glutamicum* accumulates α-ketoglutarate from d-xylose via the Weimberg pathway.

## 1 Introduction

The Gram-positive bacterium *Corynebacterium glutamicum* has a long history in the industrial production of proteinogenic amino acids. In particular l-glutamate and l-lysine are produced at million ton-scale with this microorganism (Eggeling and Bott, 2015; Lee and Wendisch, 2017). Furthermore, *C. glutamicum* strains for more than 70 biotechnologically interesting compounds such as alcohols, organic acids or polyphenols have been engineered over the last years (Becker et al., 2018; Kallscheuer et al., 2016, 2017; Vogt et al., 2016; Wieschalka et al., 2013). However, all large-scale applications for amino acid production with *C. glutamicum* use d-glucose from starch hydrolysates or d-fructose (and sucrose) from molasses and the substrate spectrum of *C. glutamicum* variants engineered for other small molecules is also for the most part limited to these hexoses (Blombach and Seibold, 2010).

**Fig. 1:**
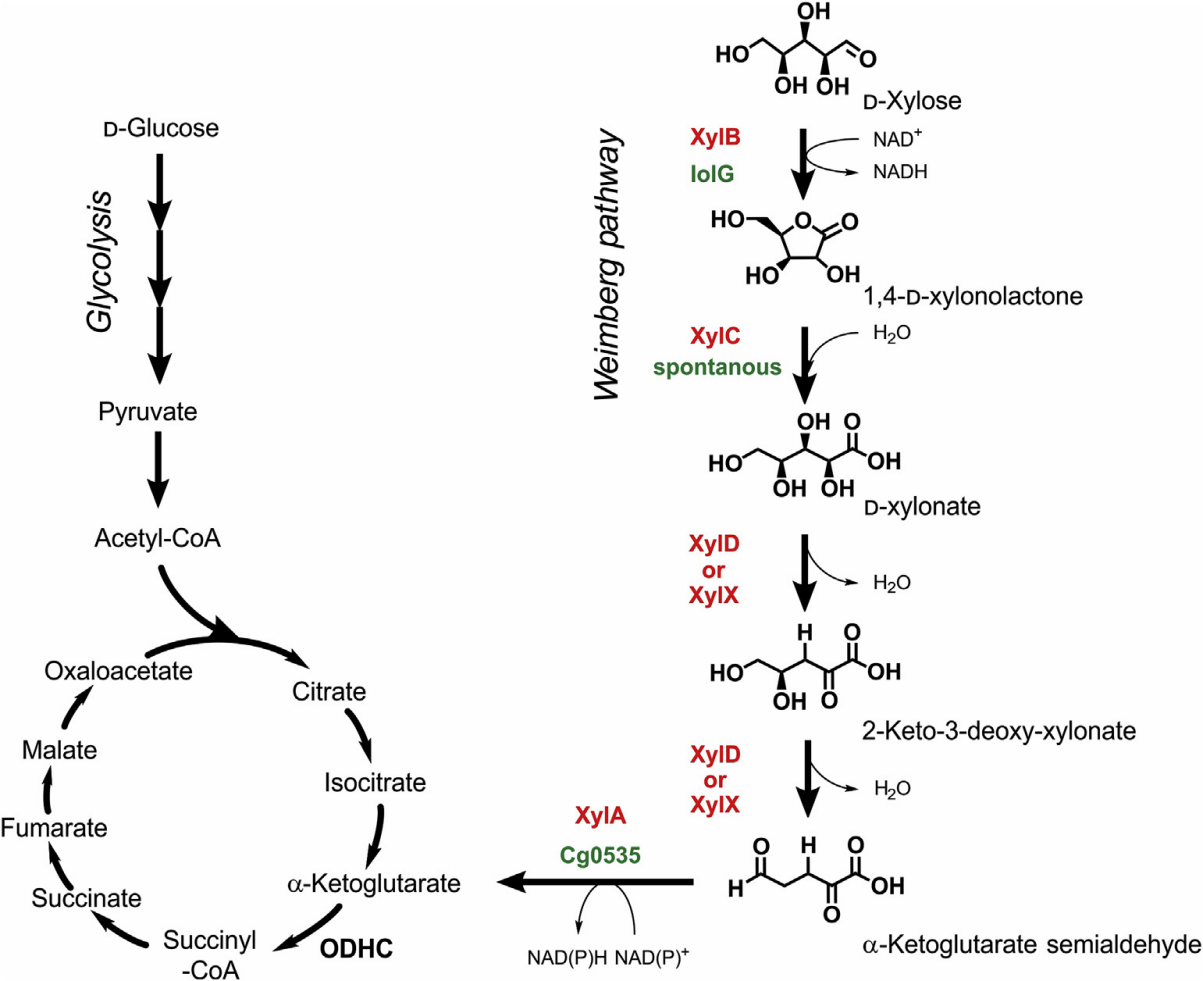
Schematic overview of the metabolic connection of the Weimberg pathway to the central carbon metabolism of *C. glutamicum*. Endogenous enzymes of *C. glutamicum* catalyzing reactions of the Weimberg pathway or spontaneous chemical reactions are highlighted in green, whereas the respective heterologous enzymes originating from *C. crescentus* are highlighted in red. Abbreviations: XylB, xylose dehydrogenase; XylC, d-1,4-xylono lactonase; XylD, d-xylonate dehydratase; XylX, 2-keto-3-deoxy-d-xylonate dehydratase; XylA, α-ketoglutarate semi aldehyde dehydrogenase; IolG, *myo*-inositol-2-dehydrogenase; KsaD, α-ketoglutarate semialdehyde dehydrogenase; ODHC, α-ketoglutarate dehydrogenase complex. (For interpretation of the references to colour in this figure legend, the reader is referred to the Web version of this article.)

More recent studies focus on engineering *C. glutamicum* for the utilization of lignocellulose-derived pentoses d-xylose and l-arabinose as *C. glutamicum* cannot naturally catabolize these sugars (Kawaguchi et al., 2006, 2008). In case of d-xylose, two different metabolic routes have been individually added to the catabolic repertoire of *C. glutamicum*. In the Isomerase pathway, d-xylose is first converted to d-xylulose by a heterologous d-xylose isomerase (encoded by *xylA* from either *Escherichia coli* or *Xanthomonas campestris*) and subsequently phosphorylated by an endogenous d-xylulokinase (encoded by *xylB*) yielding d-xylulose-5-phosphate, which can be rapidly metabolized (Kawaguchi et al., 2006; Meiswinkel et al., 2013). Several *C. glutamicum* strains, capable of utilizing d-xylose via the Isomerase pathway have been engineered for the production of succinate, ethanol, lysine, glutamate, ornithine, putrescine and 1,5-diaminopentane (Buschke et al., 2011; Jo et al., 2017; Meiswinkel et al., 2013). In contrast, functional introduction of the *xylXABCD* operon from *Caulobacter crescentus* enabled *C. glutamicum* to grow on d-xylose as sole carbon and energy source via the five-step Weimberg pathway (Radek et al., 2014). In this pathway, d-xylose is initially oxidized to 1,4-d-xylonolactone via a xylose dehydrogenase (XylB) and subsequently hydrolyzed by a d-xylonolactonase (XylC) yielding d-xylonate (Fig. 1). Two subsequent dehydration reactions, catalyzed by a d-xylonate dehydratase (XylD) and a 2-keto-3-deoxyxylonate dehydratase (XylX), lead to α-ketoglutarate semialdehyde, which is finally oxidized by an α-ketoglutarate semialdehyde dehydrogenase (XylA) to the tricarboxylic acid (TCA)-cycle intermediate α-ketoglutarate. However, *C. glutamicum* WMB1 as the first engineered strain having the Weimberg pathway allowed only for a growth rate of μ = 0.07 h^−1^ on d-xylose containing defined medium. Adaptive laboratory evolution improved d-xylose utilization by 260 % yielding the strain *C. glutamicum* WMB2_evo_ (μ_max_ = 0.26 h^−1^) (Radek et al., 2017). Genome sequencing of this strain revealed a functional loss of the transcriptional regulator IolR, which controls the expression of 22 genes for the most part believed to be involved in *myo*-inositol metabolization (Klaffl et al., 2013). Among these genes is *iolT1* encoding for the *myo*-inositol/proton symporter IolT1, which turned out to also contribute to d-xylose uptake in *C. glutamicum* (Brüsseler et al., 2018). By rationally introducing two point mutations into the IolR-binding site of the *iolT1*-promoter yielding *C. glutamicum* P_O6_ *iolT1,* this effect could be successfully mimicked. Furthermore, an endogenously encoded d-xylose dehydrogenase (IolG) contributing to the oxidation of d-xylose in *C. glutamicum* could be identified, which was subsequently employed for the carbon efficient production of d-xylonate with *C. glutamicum* (Tenhaef et al., 2018).

These studies show that the *C. glutamicum* wild type, although not capable of d-xylose utilization via the Weimberg pathway or any other catabolic strategy by nature, does already possess individual Weimberg pathway components enabling d-xylose transport and initial d-xylose oxidation. This causes one to wonder whether there are additional endogenous enzymatic activities contributing to d-xylose utilization, which would help to reduce the number of heterologous genes required for establishing the Weimberg pathway in this bacterium.

In this study, we performed an analysis of the *C. glutamicum* genome in combination with systematic reduction of the *xylXABCD* operon to identify such enzymes. Furthermore, we exploited the Weimberg pathway for the direct conversion of d-xylose to α-ketoglutarate and could show that this represents a promising strategy for the microbial production of α-ketoglutarate with *C. glutamicum*.

## 2 Materials and methods

### 2.1 Bacterial strains, plasmids, media and growth conditions

All used bacterial strains and plasmids including their characteristics and sources are listed in Table 1. *Escherichia coli* DH5α, used for cloning purposes only, was routinely cultivated on a rotary shaker (170 rpm, 37 °C) in reaction tubes with 5 mL Lysogeny Broth (LB) medium (Bertani, 1951) or on LB agar plates (LB medium with 1.8 % [wt/vol] agar). All *C. glutamicum* strains are derived from *C. glutamicum* ATCC 13032 (Abe et al., 1967) and were aerobically cultivated on a rotary shaker either in reaction tubes (170 rpm, 30 °C) or in baffled shake flasks (130 rpm, 30 °C). As cultivation medium, brain heart infusion (BHI) medium (Difco Laboratories, Detroit, USA) or defined CGXII medium (Keilhauer et al., 1993) supplemented with different d-glucose/d-xylose mixtures were used. For plasmid propagation, kanamycin was added to final concentrations of 25 μg mL^−1^ (*C. glutamicum*) or 50 μg mL^−1^ (*E. coli*). Where appropriate, the antibiotic spectinomycin was added to a final concentration of 100 μg mL^−1^. Induction of gene expression was achieved by isopropyl β-d-thiogalactoside (IPTG) supplementation to a final concentration of 1 mM. In general, growth of bacterial strains, cultivated in baffled shake flasks, was followed over time by measuring the optical density at 600 nm (OD_600_). Cultivations in the microtiter plate format were performed in Flower Plates with optodes using the microbioreactor BioLector (m2plabs, Baesweiler, Germany), enabling online determination of backscatter, pH and dissolved oxygen. BioLector cultivations were routinely inoculated to an OD_600_ of 1 and incubated at 30 °C, 1300 rpm and 80 % humidity. The total culture volume was always 1 mL and the backscatter gain was set to 15.

**Table 1:** Strains and plasmids used in this study.

Strain or plasmid	Relevant characteristics^a^	Source or reference
***C. glutamicum* strains**		
ATCC 13032 (WT)	biotin auxotroph wild-type strain	Abe et al. (1967)
P_O6_*iolT1*	Derivative of *C. glutamicum* ATCC 13032 with two point mutations in the promotor of *iolT1*, relative to the start codon at position −113 (A→G) and −112 (C→G) respectively	(Brüsseler et al., 2018)
P_O6_*iolT1 Δ*cg0535	Derivative of *C. glutamicum* P_O6_*iolT1* with in-frame deletion of cg0535 (*ksaD*)	This study
P_O6_*iolT1 ΔodhA*	Derivative of *C. glutamicum* P_O6_*iolT1* with in-frame deletion of *odhA* (cg1280)	This study
***E. coli* strains**		
DH5α	F^−^ Φ80*lac*ZΔM15 Δ(*lac*ZYA-*arg*F)U169 *rec*A1 *end*A1 *hsd*R17 (r_K_^–^, m_K_^+^) *pho*A *sup*E44 λ– *thi*-1 *gyr*A96 *rel*A1	Invitrogen (Karlsruhe, Germany)
BL21 (DE3)	F^−^*ompT hsdSB*(r_B_^−^ m_B_^−^) *gal dcm* (DE3)	Invitrogen (Karlsruhe, Germany)
***C. glutamicum* Plasmids**		
pEKEx3	Spec^r^; *C. glutamicum*/*E. coli* shuttle vector for regulated gene expression; (P_tac_, lacI^Q^, pBL1 *ori*V*Cg*, pUC18 *ori*V*Ec*)	(Gande et al., 2007)
pEKEx3-*xylXABCD*_*Cc*_-opt	Spec^r^; pEKEx3 derivative for the regulated expression of *xylXABCD*_*Cc*_ of *C. crescentus*	Radek et al. (2017)
pEKEx3-*xylXAD*_*Cc*_-opt	Spec^r^; pEKEx3 derivative for the regulated expression of *xylXAD*_*Cc*_ of *C. crescentus*	This study
pEKEx3-*xylXD*_*Cc*_-opt	Spec^r^; pEKEx3 derivative for the regulated expression of *xylXD*_*Cc*_ of *C. crescentus*	This study
pEKEx3-*xylX*_*Cc*_-opt	Spec^r^; pEKEx3 derivative for the regulated expression of *xylX*_*Cc*_ of *C. crescentus*	This study
pEKEx3-*xylD*_*Cc*_-opt	Spec^r^; pEKEx3 derivative for the regulated expression of *xylD*_*Cc*_ of *C. crescentus*	This study
pk19*mobsacB-*Δcg0535	Kan^r^; plasmid for in-frame deletion of cg0535 (*ksaD*)	This study
pk19*mobsacB-*Δ*odhA*	Kan^r^; plasmid for in-frame deletion of *odhA* (cg1280)	This study

***E. coli* Plasmids**		
pET-28b(+)	Kan^r^; Vector for overexpression of genes in *E. coli*, adding an N-terminal hexahistidine affinity tag to the synthesized protein (pBR322 *oriV*_*E.c.*_ P_T7_*lacI*)	Novagen (Darmstadt, vector, Germany)
pET-28b(+)-cg0535	Kan^r^; pET-28b(+) derivative for the regulated expression of cg0535 (*ksaD*) of *C. glutamicum*	This study

a Kan^r^; Kanamycin resistance, Spec^r^; Spectinomycin resistance.

### 2.2 Plasmid and strain construction

All enzymes were purchased from Thermo Scientific (Schwerte, Germany) whereas codon-optimized synthetic genes for expression in *C. glutamicum* were obtained from Life Technologies (Darmstadt, Germany). Oligonucleotides were synthesized by Eurofins genomics (Ebersfeld, Germany) and are listed in Table 2. For molecular cloning work, standard protocols, e.g. PCR and Gibson were used (Gibson et al., 2009; Sambrook and Russel, 2001). Verification of the constructed plasmids was performed either by restriction analysis or colony PCR. DNA sequencing was conducted at Eurofins Genomics (Ebersberg, Germany). *E. coli* DH5α was routinely transformed using the RbCl-method, whereas *C. glutamicum* was always transformed by electroporation followed by an additional heat shock at 46 °C for 6 min (Eggeling and Bott, 2005; Hanahan, 1983). In-frame deletion of *odhA* and cg0535 (*kasD*) was performed by two-step homologues recombination using the plasmids pk19*mobsacB*-Δ*odhA* and pk19*mobsacB*-Δcg0535 as previously described (Schäfer et al., 1994).

**Table 2:** Oligonucleotides used in this study.

Name	DNA Sequence (5′- 3′)	
**Construction of pEKEx3-*xylXAD***_**Cc**_**-opt**		
pe3_check fw	CGGCGTTTCACTTCTGAGTTCGGC	
pe3_check rev	GATATGACCATGATTACGCCAAGC	
pe3_xylXAD_xylX_fw	GCCAAGCTTGCATGCCTGCATAACTAGTATAAGGAGATATAGATATGG	
pe3_xylXAD_xylX_rev	TTATACTAGCTTATTACAGCAGGCCACG	
pe3_xylXAD_xylA_fw	GCTGTAATAAGCTAGTATAAGGAGATATAGATATGAC	
pe3_xylXAD_xylA_rev	TTATACTAGCTTATTAGGACCAGGAGTAGG	
pe3_xylXAD_xylD_fw	GTCCTAATAAGCTAGTATAAGGAGATATAGATATGC	
pe3_xylXAD_xylD_rev	CTGTAAAACGACGGCCAGTGTTATTAGTGGTTGTGGCG	
**Construction of pEKEx3-*xylXD***_**Cc**_**-opt**		
pe3_check fw	CGGCGTTTCACTTCTGAGTTCGGC	
pe3_check rev	GATATGACCATGATTACGCCAAGC	
pe3_xylXD_xylX_fw	GCCAAGCTTGCATGCCTGCAGCTAGTATAAGGAGATATAGATATGGGCGTGTCCGAGTTC	
pe3_xylXD_xylX_rev	CGGAGCGCATATCTATATCTCCTTATACTAGCTTATTACAGCAG	
pe3_xylXD_xylD_fw	AGATATAGATATGCGCTCCGCACTGTCC	
pe3_xylXD_xylD_rev	CTGTAAAACGACGGCCAGTGTTATTAGTGGTTGTGGCGTGGC	
**Construction of pk19*mobsacB*- Δcg0535 (*ksaD*)**		
rsp	CACAGGAAACAGCTATGACCATG	
univ	CGCCAGGGTTTTCCCAGTCACGAC	
cg0535_seq_fw	AATCCACTTCTCTTGGTGTCATCGT	
cg0535_seq_rev	CTTCGAGGACGCGAGTATTCATATT	
cg0535_fw_fw	TGCATGCCTGCAGGTCGACTATCTACTCCCCAGAGGTTATCG	
cg0535_fw_rev	CCCATTTATTTGCGGTTGCGGTGATCATG	
cg0535_rev_fw	CGCAACCGCAAATAAATGGGCTGTACCTC	
cg0535_rev_rev	TTGTAAAACGACGGCCAGTGCGCTAGATTTAGGCCTTG	
**Construction of pk19*mobsacB*-Δ*odhA* (cg1280)**		
rsp	CACAGGAAACAGCTATGACCATG	
univ	CGCCAGGGTTTTCCCAGTCACGAC	
odhA check fw	GAAGCACACTTGTTTAGTGG	
odhA check rev	CCCGTAGAGATCGGCTGGGT	
odhA fw_fw	TGCATGCCTGCAGGTCGACTCCATCGCCGCCATCCCTG	
odhA fw_rev	TAAGCTGCTTCTCAGTACTAGCGCTGCTCACGG	
odhA rev_fw	CGCTAGTACTGAGAAGCAGCTTATCGAC	
odhA rev_rev	TTGTAAAACGACGGCCAGTGTCCATTATCGTAGGTGATG	
**Construction of pET-28b(+)-cg0535**		
pET16b_fw	GATCCCGCGAAATTAATACG	
pET16b_rv	CAAGACCCGTTTAGAGGCCCC	
cg0535_fw	CTGGTGCCGCGCGGCAGCCACATGATCACCGCAACCGC	
cg0535_rev	AAGCTTGTCGACGGAGCTCGTTAACGGTCTATTTCCCGAGG	

### 2.3 Microbial production of α-ketoglutarate

For initial biomass formation, all constructed *C. glutamicum* strains were cultivated in 50 mL BHI medium with 10 g/l d-glucose in 500 mL baffled shake flasks at 130 rpm and 30 °C on a rotary shaker. Cells were harvested by centrifugation at 4000 rpm for 10 min, resuspended in defined CGXII medium with either 4 % d-glucose or a 1 % d-glucose/3 % d-xylose mixture and then further cultivated for 40 h at 130 rpm and 30 °C on a rotary shaker. For α-ketoglutarate production, defined CGXII medium with either 4 % d-glucose or a 1 % d-glucose/3 % d-xylose mixture was inoculated to an OD_600_ of 4. If appropriate, gene expression was induced by adding IPTG to a final concentration of 1 mM.

### 2.4 Heterologous expression of Cg0535 in *E. coli* and protein purification

The plasmid pET-28b(+)-cg0535 was transformed into *E. coli* BL21 for heterologous gene expression of cg0535. Cultivations for this purpose were performed in 10 mL 2xYT medium in baffled shake flasks for 15 h at 37 °C and 130 rpm on a rotary shaker. 1 mL of this culture was used to inoculate an expression culture in 100 mL 2xYT medium with 50 mg L^−1^ kanamycin and cultivated at 37 °C and 130 rpm. At an optical density of OD_600_ = 1.5, gene expression was induced by the addition of 0.5 mM IPTG and then further incubated at 18 °C and 130 rpm for 18 h. Cells were harvested by centrifugation for 30 min at 6000 rpm and the cell-free supernatant was discarded. Cell pellets were routinely stored at −80 °C if not further processed the same day. In order to avoid protein degradation, all subsequent steps for protein isolation were performed at 4 °C. Frozen cell pellets were first thawed on an ice-water mixture and resuspended in 15 mL lysis buffer (50 mM Tris-HCl pH 7.6, 100 mM NaCl, 10 mM Imidazole, 5 % Glycerin and 1 mM DTT). Crude cell extracts were obtained by using a Branson Sonifier 250 (intensity, 7; duty cycle, 40 %, 6 min; Branson Ultrasonics, Danbury, USA). After removal of the cellular debris by two centrifugation steps (30 min at 6000 rpm and 45 min at 50,000 rpm) Cg0535 was purified from the protein fraction by affinity chromatography using a GE Äkta pure chromatography system (GE Healthcare Life Sciences, Chicago, USA).

### 2.5 Kinetic characterization of KsaD (Cg0535)

In all dehydrogenase assays performed, the initial NAD(P)H generation due to KsaD-mediated α-ketoglutarate semialdehyde oxidation was monitored at 340 nm and 30 °C using an Shimadzu UV-1601 Spectrophotometer (Kyoto, Japan). The enzyme assays contained 0–5 mM α-ketoglutarate semialdehyde (FCH Group, Chernigiv, Ukraine, supplied by AKos Consulting & Solutions Deutschland GmbH, Steinen, Germany), 5 mM NAD(P)^+^, 100 mM Potassium phosphate, pH 7.5. Assays were linear over time and proportional to the protein concentration used.

### 2.6 Quantification of d-xylose

For quantification of d-xylose, a commercial enzyme assay kit was used according to the manufacturer's instructions (Xylose Assay Kit, Megazymes, Wickow, Ireland). A set of different d-xylose concentrations served as external standards.

### 2.7 HPLC analysis

Identification and quantification of metabolites was performed using a High Performance Liquid Chromatography (HPLC) 1260 Infinity system (Agilent, Waldbronn, Germany). Separation was achieved by using an Organic acid H^+^ column (8 %, 300 mm by 7.80 mm; Phenomenex, Torrance, CA, USA) at 80 °C with an isocratic elution program using 5 mM sulfuric acid. For detection of organic acids and d-glucose, a diode array detector (DAD) at 210 nm or a refraction index (RI) detector was used, respectively. Data acquisition and analysis was performed using the Agilent OpenLAB Data Analysis - Build 2.200.0.528 software (Agilent, Waldbronn, Germany).

## 3 Results

### 3.1 Identification of an endogenous α-ketoglutarate semialdehyde dehydrogenase activity

**Fig. 2:**
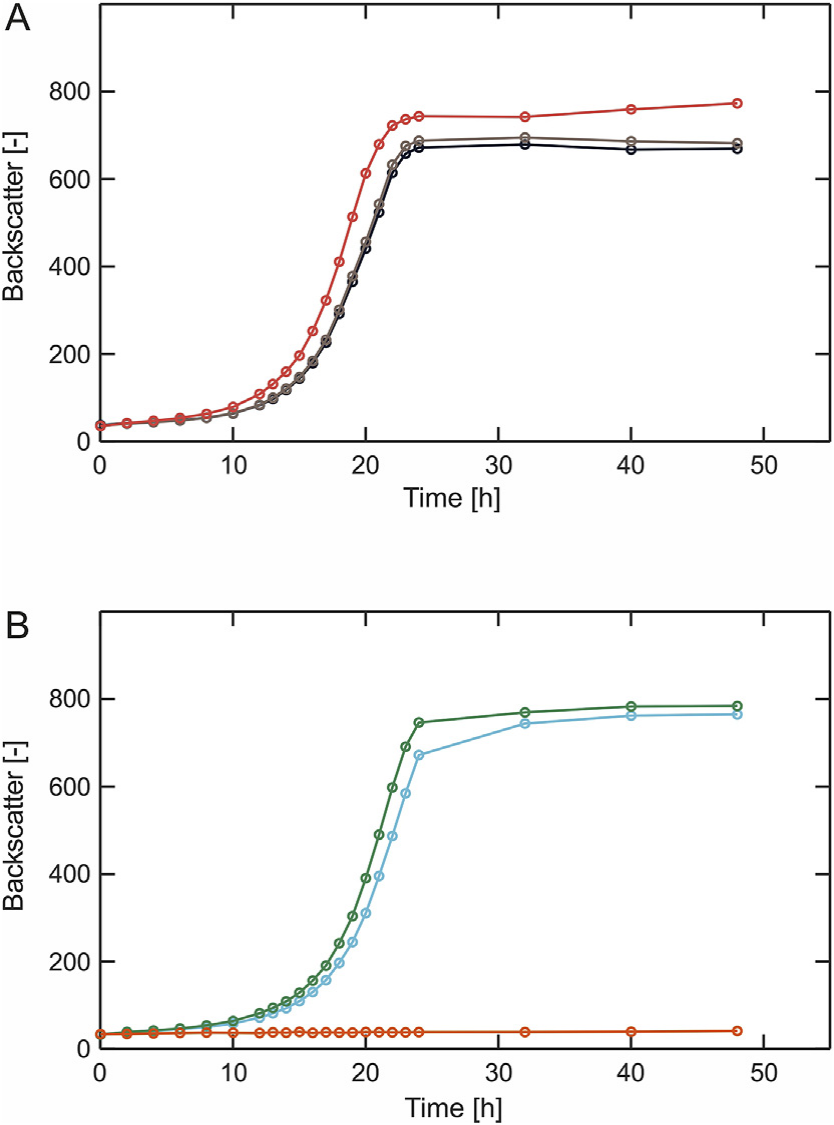
Microbioreactor cultivations of *C. glutamicum* strains engineered for d-xylose utilization via the Weimberg pathway. **(A)***C. glutamicum* P_O6_*iolT1* pEKEx3-*xylXABCD*_*Cc*_*-opt (black), C. glutamicum* P_O6_*iolT1* pEKEx3-*xylXAD*_*Cc*_*-opt* (*brown*) and *C. glutamicum* P_O6_*iolT1* pEKEx3-*xylXD*_*Cc*_*-opt (red)*; **(B)***C. glutamicum* P_O6_*iolT1* pEKEx3-*xylX*_*Cc*_*-opt (cyan), C. glutamicum* P_O6_*iolT1* pEKEx3-*xylD*_*Cc*_*-opt* (*green*) and *C. glutamicum* P_O6_*iolT1* pEKEX3 *(orange)*. All strains were cultivated in a BioLector microbioreactor system using defined CGXII medium with 40 g L^−1^d-xylose as sole carbon and energy source. All data represent mean values from three biological replicates. (For interpretation of the references to colour in this figure legend, the reader is referred to the Web version of this article.)

Presence of the endogenous dehydrogenase IolG oxidizing d-xylose to 1,4-d-xylonolactone and the observation that hydrolyzation of this lactone can occur spontaneously, indicates that heterologous expression of the xylose dehydrogenase (encoded by *xylB*) and the d-xylonolactonase (encoded by *xylC*) from *C. crescentus* might not be required for establishing the Weimberg pathway in *C. glutamicum*. Since reduction of the Weimberg pathway encoding operon has not been tried yet, a synthetic operon comprised of codon-optimized genes for 2-keto-3-desoxyxylonate dehydratase (*xylX*), xylonate dehydratase (*xylD*) and the α-ketoglutarate semialdehyde dehydrogenase (*xylA*), all originating from *C. crescentus*, was constructed. The resulting pEKEx3-*xylXAD*_*Cc*_-opt plasmid was then transferred to *C. glutamicum* P_O6_ *iolT1*, which is characterized by deregulation of the *myo*-inositol/proton symporter gene *iolT1.* Growth of the resulting strain *C. glutamicum* P_O6_ *iolT1* pEKEx3-*xylXAD*_*Cc*_-opt was compared to that of *C. glutamicum* P_O6_ *iolT1* pEKEx3-*xylXABCD*_*Cc*_-opt bearing the complete *xylXABCD* operon from *C. crescentus* (Fig. 2A). Surprisingly, growth of both strains was indistinguishable (μ_max_ = 0.26 ± 0.006 h^−1^, μ_max_ = 0.26 ± 0.004 h^−1^, respectively), indicating that heterologous expression of the xylose dehydrogenase (encoded by *xylB*) and the xylonolactonase (encoded by *xylC*) is neither necessary nor beneficial for growth of *C. glutamicum*.

Motivated by these results, we performed a genome-wide search based on sequence similarity to identify genes potentially encoding for enzymes with XylX-, XylD- or XylA-activity in *C. glutamicum* ATCC 13032. These analyses suggested that the gene cg0535 could encode for an enzyme having a α-ketoglutarate semialdehyde dehydrogenase activity. However, the predicted protein Cg0535 shares only 25 % sequence identity with XylA of *C. crescentus*. For a better assessment, secondary structures of Cg0535 and XylA were calculated and aligned using PROMALS3D (PROfile Multiple Alignment with predicted Local Structures and three-dimensional constraints) (Supplementary Fig. S1) (Pei et al., 2008). This *in silico* analysis revealed a striking resemblance between both proteins with regard to their secondary structure triggering further investigations. To the best of our knowledge, nothing about regulation and expression of cg0535 in *C. glutamicum* is known. Nonetheless, *C. glutamicum* P_O6_ *iolT1* pEKEx3-*xylXD*_*Cc*_-opt with a further reduced operon was constructed to find out whether heterologous expression of *xylA* from *C. crescentus* is required for establishing the Weimberg pathway in *C. glutamicum*. Comparative cultivation of *C. glutamicum* P_O6_ *iolT1* pEKEx3-*xylXD*_*Cc*_-opt and *C. glutamicum* P_O6_ *iolT1* pEKEx3-*xylXAD*_*Cc*_-opt revealed that *C. glutamicum* indeed does have an endogenous α-ketoglutarate semialdehyde dehydrogenase as both strains exhibited the same growth rate (μ_max_ = 0.26 ± 0.008 h^−1^, μ_max_ = 0.26 ± 0.006 h^−1^, respectively) (Fig. 2A). Subsequently, cg0535 was deleted in the genome of *C. glutamicum* P_O6_ *iolT1*, yielding *C. glutamicum* P_O6_ *iolT1 Δ*cg0535. After transformation of this strain with pEKEx3-*xylXD*_*Cc*_-opt, the resulting strain *C. glutamicum* P_O6_ *iolT1* Δcg0535 pEKEx3-*xylXD*_*Cc*_-opt was compared to its parent strain *C. glutamicum* P_O6_ *iolT1* pEKEx3-*xylXD*_*Cc*_-opt. These experiments showed that deletion of cg0535 completely abolished growth of *C. glutamicum* P_O6_ *iolT1* Δcg0535 pEKEx3*xylXD*_*Cc*_-opt confirming that Cg0535 indeed has α-ketoglutarate semialdehyde dehydrogenase activity (data not shown). The inability of the cg0535 deletion mutant to grow on d-xylose also indicates that Cg0535 appears to be the only endogenous enzyme of *C. glutamicum* significantly contributing to α-ketoglutarate semialdehyde oxidation, at last under the cultivation conditions tested.

With the aim to characterize Cg0535 in more detail, the cg0535 gene was isolated from the genome of *C. glutamicum* ATCC 13032 by PCR and cloned into the pET-28b(+)-vector for heterologous expression in *E. coli* BL21 (DE3). Gene expression in *E. coli* at 100 mL-scale and subsequent protein purification by affinity chromatography yielded 0.6 mg Cg0535 protein. Subsequently, we performed *in vitro* dehydrogenase assays using α-ketoglutarate semialdehyde as substrate and NAD or NADP as cofactors to determine selected kinetic parameters of Cg0535. These *in vitro* experiments confirmed the assumed α-ketoglutarate semialdehyde dehydrogenase activity of this enzyme and furthermore revealed a preference for the cofactor NAD as the calculated specific activity (U mg^−1^) with NAD was three times higher compared to the activity with NADP (51.8 μmol min^−1^ mg^−1^ and 15.8 μmol min^−1^ mg^−1^, respectively) (Supplementary Fig. S2). Therefore, depending on the cofactor used, different Michaelis constants (K_m_) for α-ketoglutarate semialdehyde could be calculated (NAD, 0.87 mM; NADP, 0.21 mM). Considering these findings, we would like to introduce the designation *ksaD* (α-ketoglutarate semialdehyde dehydrogenase) for cg0535 of *C. glutamicum*.

### 3.2 Expression of *xylD* or *xylX* enables growth on d-xylose

Analysis of the *C. glutamicum* genome did not identify any hitherto unknown dehydratases potentially catalyzing the two subsequent dehydration reactions of the Weimberg pathway. Noteworthy, enzyme assays conducted with the dehydratases XylX and XylD of *C. cresentus* showed that both dehydratases accept d-xylonate as substrate (Dahms and Donald, 1982). Since both dehydratase substrates d-xylonate and 2-keto-3-deoxy-xylonate of the Weimberg pathway, are chemically quite similar, it makes one wonder why two separate enzymes appear to be necessary (Fig. 1). Unfortunately, no experimental data shedding more light on this interesting aspect are available for the enzymes of *C. crescentus*. However, a conducted comparison of both enzymes as part of this study revealed only a low sequence identity (18 %) and an analysis using PROMALS3D suggested two very different secondary structures (data not shown). Nevertheless, driven by curiosity, the plasmids pEKEx3-*xylX*_*Cc*_-opt and pEKEx3-*xylD*_*Cc*_-opt were constructed and individually introduced into *C. glutamicum* P_O6_ *iolT1*. The resulting strains *C. glutamicum* P_O6_ *iolT1* pEKEx3-*xylX*_*Cc*_-opt and *C. glutamicum* P_O6_ *iolT1* pEKEx3-*xylD*_*Cc*_-opt were compared with regard to growth to *C. glutamicum* P_O6_ *iolT1* pEKEx3 (Fig. 2B). As a result, both strains expressing either *xylX* or *xylD* could grow on this defined medium with d-xylose as sole carbon and energy source, whereas *C. glutamicum* P_O6_ *iolT1* could not. The growth rates of *C. glutamicum* P_O6_ *iolT1* pEKEx3-*xylX*_*Cc*_-opt and *C. glutamicum* P_O6_ *iolT1* pEKEx3-*xylD*_*Cc*_-opt were identical (μ_max_ = 0.25 ± 0.006 h^−1^, μ_max_ = 0.25 ± 0.004 h^−1^, respectively). Apparently, both dehydratases can complement for each other in *C. glutamicum* and heterologous expression of either *xylX* or *xylD* from *C. crescentus* is sufficient for enabling d-xylose utilization via the Weimberg pathway in *C. glutamicum* P_O6_ *iolT1*.

### 3.3 α-ketoglutarate synthesis via the Weimberg pathway

**Fig. 3:**
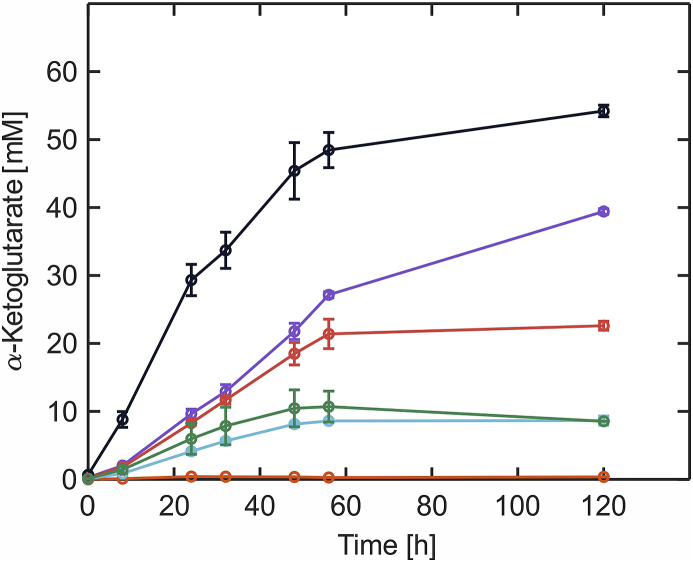
Accumulation of α-ketoglutarate during shake flask cultivations of different *C. glutamicum* strains in defined CGXII medium supplemented with either 40 g L^−1^d-glucose or a mixture of 10 g L^−1^d-glucose and 30 g L^−1^d-xylose. *C. glutamicum* P_O6_*iolT1* (*orange*, 40 g L^−1^d-glucose), *C. glutamicum* P_O6_*iolT1 ΔodhA* (*purple*, 40 g L^−1^d-glucose), *C. glutamicum* P_O6_*iolT1 ΔodhA* pEKEx3-*xylXABCD*_*Cc*_*-opt* (*black*, 10 g L^−1^d-glucose and 30 g L^−1^d-xylose), *C. glutamicum* P_O6_*iolT1 ΔodhA* pEKEx3-*xylXD*_*Cc*_*-opt* (*red*, 10 g L^−1^d-glucose and 30 g L^−1^d-xylose), *C. glutamicum* P_O6_*iolT1 ΔodhA* pEKEx3-*xylX*_*Cc*_*-opt* (*cyan*, 10 g L^−1^d-glucose and 30 g L^−1^d-xylose), *C. glutamicum* P_O6_*iolT1 ΔodhA* pEKEx3-*xylD*_*Cc*_*-opt* (*green*, 10 g L^−1^d-glucose and 30 g L^−1^d-xylose). The data represent mean values and standard deviations obtained from three independent cultures. (For interpretation of the references to colour in this figure legend, the reader is referred to the Web version of this article.)

The Weimberg pathway represents a shortcut to the biotechnologically interesting TCA-cycle intermediate α-ketoglutarate without loss of carbon as compared to α-ketoglutarate synthesis starting from d-glucose (Fig. 1) (Jo et al., 2012). Nevertheless, microbial production of this dicarboxylic acid from d-xylose via the Weimberg pathway with *C. glutamicum* has not been investigated, yet. Within the TCA-cycle of *C. glutamicum*, the large multienzyme α-ketoglutarate dehydrogenase complex (ODHC) is responsible for the oxidative decarboxylation of α-ketoglutarate (Usuda et al., 1996; Bott, 2007). ODHC is comprised of three subunits: E1o (α-ketoglutarate decarboxylase, OdhA), E2 (dihydrolipoamide acetyl/succinyl-transferase, AceF) and E3 (dihydrolipoamide dehydrogenase, Lpd). It could be shown previously, that deletion of *odhA* results in the accumulation of α-ketoglutarate (Asakura et al., 2007). With the aim of establishing microbial α-ketoglutarate production from d-xylose via the Weimberg pathway in *C. glutamicum*, *odhA* was also deleted in *C. glutamicum* P_O6_ *iolT1*. Initially, the resulting strain *C. glutamicum* P_O6_ *iolT1 ΔodhA* was cultivated in defined CGXII medium supplemented with 40 g L^−1^ d-glucose as the sole carbon and energy source to find out if this is able to overproduce α-ketoglutarate from this hexose. Within 120 h, this strain accumulated 5.76 ± 0.06 g L^−1^ (39.43 ± 0.4 mM) α-ketoglutarate in the supernatant (Fig. 3). In contrast, the parent strain *C. glutamicum* P_O6_ *iolT1* without deletion of *odhA* accumulated only 0.05 ± 0.00 g L^−1^ (0.37 ± 0.03 mM) α-ketoglutarate. Subsequently, *C. glutamicum* P_O6_ *iolT1 ΔodhA* was transformed with pEKEx3-*xylXABCD*_*Cc*_-opt to find out whether the resulting strain accumulates more α-ketoglutarate in d-glucose/d-xylose mixtures. Noteworthy, deletion of *odhA* interrupting the TCA-cycle renders cultivation on d-xylose as sole carbon and energy source impossible. Here, *C. glutamicum* P_O6_ *iolT1 ΔodhA* pEKEx3-*xylXABCD*_*Cc*_-opt accumulated 7.92 ± 0.13 g L^−1^ (54.21 ± 0.86 mM) α-ketoglutarate in the supernatant when cultivated in defined CGXII medium with 10 g L^−1^ d-glucose and 30 g L^−1^ d-xylose (Fig. 3). In comparison to cultivation of *C. glutamicum* P_O6_ *iolT1 ΔodhA* in defined medium containing only d-glucose, the product titer could be increased 1.5-fold. Motivated by these findings, *C. glutamicum* P_O6_ *iolT1 ΔodhA* pEKEx3-*xylX*_*Cc*_-opt and *C. glutamicum* P_O6_ *iolT1 ΔodhA* pEKEx3-*xylD*_*Cc*_-opt were also constructed and characterized with regard to their α-ketoglutarate production capabilities on d-glucose/d-xylose mixtures. Interestingly, both strains accumulated much less α-ketoglutarate in the supernatant compared to the strain with the full *xylXABCD*-operon (1.27 ± 0.1 g L^−1^ (8.71 ± 0.7 mM) and 1.26 ± 0.0 g L^−1^ (8.62 ± 0.0 mM, respectively). This was somewhat surprising, as these results hint towards a limitation of the flux through the Weimberg pathway during product formation, which was not observable during growth experiments with *C. glutamicum* strains without *odhA*-deletion. Subsequent construction and characterization of *C. glutamicum* P_O6_ *iolT1 ΔodhA* pEKEx3-*xylXD*_*Cc*_-opt, bearing the plasmid for expression of both dehydratase genes from *C. crescentus*, supports the hypothesis of a restricted flux through the Weimberg pathway in this strain background because an increased α-ketoglutarate concentration of 3.30 ± 0.09 g L^−1^ (22.61 ± 0.65 mM) could be determined in the supernatant.

## 4 Discussion

Functional introduction of a pathway from another organism or implementation of a novel synthetic pathway usually means addition of new enzymatic activities to the catalytic repertoire of the respective host organism. However, sometimes the “new” enzymes have overlapping substrate specificities with endogenous enzymes rendering their introduction unnecessary. This could be already shown for *C. glutamicum* R and *C. glutamicum* ATCC 13032 when establishing the two-step Isomerase pathway for d-xylose utilization as both strains already have a xylulokinase (XylB) and thus only require a heterologous gene encoding for a xylose isomerase (Kawaguchi et al., 2006). In case of *Pseudomonas* sp., it could be demonstrated that the periplasmic glucose dehydrogenase (Gcd) also contributes to d-xylose utilization via the Weimberg pathway (Köhler et al., 2015; Meijnen et al., 2009). Similarly, an enzymatic study not directly connected to growth on d-xylose revealed that the two endogenous *myo*-inositol dehydrogenases IolG1 and IolG2 of *Lactobacillus casei* BL23 can convert d-xylose to d-xylonate similar to IolG of *C. glutamicum* (Aamudalapalli et al., 2018).

The endogenous α-ketoglutarate semialdehyde dehydrogenase (KsaD) of *C. glutamicum* discovered in the context of this study is characterized by a high specific activity (51.8 μmol min^−1^ mg^−1^), which is comparable to that of the enzyme with the same activity in *P. putida* (53 μmol min^−1^ mg^−1^) (Adams and Rosso, 1967). In the genome of *C. glutamicum*, the open reading frame of *ksaD* overlaps with that of cg0536 encoding for a putative 5-dehydro-4-deoxyglucarate dehydratase. This finding hints towards a potential role of KsaD in a putative oxidative pathway for the utilization of sugar acids such as d-galacturonic acid or d-glucuronic acid as theses pathways require α-ketoglutarate semialdehyde dehydrogenase- and 5-dehydro-4-deoxyglucarate dehydratase activities (Richard and Hilditch, 2009; Pick et al., 2016). It sounds reasonable that *C. glutamicum* has such a catabolic pathway as these sugar derivatives typically to be found in pectin-rich fruits and vegetables such as grapes, apples, bean sprouts should be readily available in the natural habitat of this soil bacterium (Li et al., 2016).

Heterologous expression of either *xylX* or *xylD* in *C. glutamicum* P_O6_ *iolT1* enables growth in d-xylose containing media, indicating that both dehydratases from *C. crescentus* catalyze both dehydration reactions of the Weimberg pathway in *C. glutamicum*. In contrast, a *P. putida* S12 strain equipped with the Weimberg pathway from *C. crescentus* inevitably requires the expression of *xylD* whereas heterologous expression of *xylX* alone is not sufficient for enabling growth on d-xylose (Meijnen et al., 2009). In this case, it was assumed that the endogenous dehydratase PP2836 of *P. putida* S12 exhibiting 57 % sequence identity to XylX from *C. crescentus* renders heterologous *xylX* expression unnecessary. However, this is somewhat puzzling as it would mean that the two dehydratases from *C. crescentus* cannot complement for each other. Unfortunately, the importance of having both dehydratase has not been studied in *C. crescentus* as the natural source of both enzymes yet. A detailed kinetic characterization of both dehydratases could shed more light on this important aspect. Noteworthy in this context, the archaeon *Haloferax volcanii*, naturally having the Weimberg pathway, requires the activity of both dehydratases (HVO_B0038A and HVO_B0027) for growth on d-xylose containing media (Johnsen et al., 2009).

In our experiments, microbial synthesis of α-ketoglutarate from a d-glucose/d-xylose mixture with engineered *C. glutamicum* strains having the Weimberg pathway turned out to be more beneficial for product formation compared to cultivations using d-glucose as only substrate. This could be a direct consequence of the carbon efficiency of the Weimberg pathway offering a theoretical product yield of 100 %. In contrast, α-ketoglutarate synthesis from d-glucose is always accompanied by loss of carbon as CO_2_ during isocitrate oxidation in the TCA-cycle, which eventually only allows for a maximum theoretical yield of 83 %. However, we could observe that reduction of the *xylXABCD*-operon also reduced final product concentrations in the constructed *odhA*-deletion strains. At this stage, we can only speculate that deletion of *odhA*, necessary for the accumulation of significant amounts of α-ketoglutarate, causes this effect as this is the only genetic difference to the other d-xylose consuming *C. glutamicum* strains evaluated in the context of *xylXABCD*-operon reduction. However, this indicates that heterologous expression of the whole pentacistronic *xylXABCD*-operon might not be necessary for growth of *C. glutamicum* in d-xylose containing defined medium, but is beneficial for product formation via the Weimberg pathway, especially in more engineered strains.

## 5 Conclusions

Reduction of the Weimberg pathway encoding operon from *C. crescentus* revealed that sole expression of *xylX* (2-keto-3-deoxy-xylonate-dehydratase) or *xylD* (xylonate dehydratase) is sufficient for establishing this five-step pathway in *C. glutamicum*. Reason for this is that *C. glutamicum* is already equipped with two dehydrogenases conferring the capacity to oxidize d-xylose and α-ketoglutarate semialdehyde. A lactonase converting 1,4-d-xylonolactone to d-xylonate is not required as hydrolyzation of this lactone can occur spontaneously. Conducted experiments employing the carbon efficient Weimberg pathway for the microbial synthesis of α-ketoglutarate indicate that d-xylose might represent a more suitable substrate for the production of this organic acid compared to d-glucose.
