# Supplementary material for: Alone at last! – Heterologous expression of a single gene is sufficient for establishing the five-step Weimberg pathway in Corynebacterium glutamicum
Source: Metab Eng Commun. 2019 Apr 10;9:e00090. doi: 10.1016/j.mec.2019.e00090 (PMC6475665; doi:10.1016/j.mec.2019.e00090)
Supplement: Supplementary [file mmc1.pdf]

Alone at last! – Heterologous expression of a single gene is sufficient for establishing the five-step Weimberg pathway in *Corynebacterium glutamicum*

Christian Brüsseler, Anja Späth, Sascha Sokolowsky and Jan Marienhagen*

Institute of Bio- and Geosciences, IBG-1: Biotechnology,

Forschungszentrum Jülich GmbH, Jülich D-52425, Germany

*Corresponding author

Dr. Jan Marienhagen

E-mail j.marienhagen@fz-juelich.de

Phone +49 2461 61 2843

Colored PROMALS3D alignment (sequences in aligned order)

```
Conservation:           9           9 99 99          9               9   99   9  99       9999
XylA                 1  MTD----TLRHYIGGERVAAD-APAESLNPSNTNDVVAKVPMGGQAEVDAAVDAARKAFPAWADASPEVR   65
Cg0535               1  MITATALHGCSLIDGEWVAGKNGEITGFDPRTNASLNPSYSLANSAQLRAATTSAKRAFESYRLTTPEVR   70
Consensus_aa:           Mhs....h..phIsGE.VAtc.t.hpthsPpsssslssphshtspAplcAAhstA++AF.t@..hoPEVR
Consensus_ss:                     eee  eee      eeeee     eeeeee    hhhhhhhhhhhhhhhhhhh   hhhh


Conservation:            9 99     9 9 9  9      9 9       99  9       99                9
XylA                66  SDLLDKVGSTIIARSADIGRLLAREEGKTLAEGIGETVRAGRIFKYFAGEALRRHGQNLEST-RPGVEIQ  134
Cg0535              71  ADFLDSIADNIDALSGEIVQRASLETGLGTTRLTGEVARTSNQLRLFAETVRSGQFHRVRIERGPRIDLR  140
Consensus_aa:           tDhLDpltssI.A.St-Isp.ht.EpGbshhc.hGEhhRhtpbh+hFA.ph.p.p.pplc.p..P.l-lp
Consensus_ss:           hhhhhhhhhhhhhhhhhhhhhhhhhh   hhhhhhhhhhhhhhhhhhhhhhhhh   eee      eeee


Conservation:                 9        999 9        9 999 9  99 9     9 99                 999
XylA               135  TYRQAVGVYGLITPWNFPIAI--PAWKAAPALAFGNTVVIKPAGPTPATANVLADIMAEC----GAPAGV  198
Cg0535             141  QRQVPLGPVAVFGASNFPVAFSTAGGDTASALAAGCPVVFKAHNAHPGTAELVGQAVRGAVEKHEFDAGV  210
Consensus_aa:           p.p.slGshtlhss.NFPlAh..st.chAsALAhGssVVhKshsshPtTAplltphh..t.....hsAGV
Consensus_ss:           eeee   eeeeee    hhhhhhhhhhhhhhhhh  eeeee     hhhhhhhhhhhhhhhhh      e


Conservation:           99   99    9  9          9999   9      9  9     9  99   99  9   9  99
XylA               199  FNMLFGR-GSMGDALIKHKDVDGVSFTGSQGVGAQVAAAAVARQ--ARVQLEMGGKNPLIVLDDA--DLE  263
Cg0535             211  FNLVYGRGVEIGQELAADPNITAIGFTGSRQGGLALSQTAFSRPVPVPVFAEMSATNPVFVFPGALADLD  280
Consensus_aa:           FNhl@GR.sphGp.Lh.c.slstltFTGSp.sGh.lt.hAhtR...h.VbhEMttpNPlhVhssA..DL-
Consensus_ss:           eeee   hhhhhhhhhh     eeeee  hhhhhhhhhhhhhhh ehhhhhe      eee   hhh hh


Conservation:                            99 99         9   9 9999 9 9
XylA               264  R---AVAIALDGSFFATGQRCTASSRLIVQDGIH-DKFVALLAEKVAALRVGDALDPNTQIGPAVSEDQM  329
Cg0535             281  ASSSLAEAFTASVTGSSGQLCTKPGLVFIPRGVVGDAFVALVAAKFKETTGQTM---------LTQGIAQ  341
Consensus_aa:           ....hh.hhhstsh.toGQ.CT.st.lhl.cGlh.D.FVALlA.Kh..hps.sh.........hhp...b
Consensus_ss:           hhhhhhhhhhhhhhhh   eeee eeeeee  hhhhhhhhhhhhhhh                   hhhh


Conservation:                    99         9             9                 99 99      9  9
XylA               330  ETSYRYIDIAASEGGRVVTGGDRIKLDNPGWYVRPTLIADT---QAGMRINNEEVFGPVASTIRVKSYEE  396
Cg0535             342  AWQRGVDNLAAQPSVKILAQGTPG---DGENAPGPVVFESDVQALLNNVVLQEEIFGAASLVVRYDSPDQ  408
Consensus_aa:           .hp..h.slAAp.ts+llh.Gs.....ss..hs.Phlh.ss...bhs..l.pEElFGsht.hlRhcS.-p
Consensus_ss:           hhhhhhhhhhhh   eeee           eeeeeeee          eeeee     eeeeeee  hhh


Conservation:               99  9  9 9 9                    99         9          9     9
XylA               397  ALEIANGVEFGLSAGIATTS--LKHARHFQRYAR--AGMTMVNLATAG---VDYHVPFGGTKSSSYGA-R  458
Cg0535             409  LHQVANSLEGQLTATIHASQDDFQEVSKLIPLLEDLAGRVLYGGWPTGVEVGHTVIHGGPYPATSNAQST  478
Consensus_aa:           hhplANtlE..LoAsIhhop..hpchp+hb.hhc..AG.hhhs.hshG...schhl..Gsh.toS.t..p
Consensus_ss:           hhhhhh     eeeeee   hhhhhhhhhhhhhh    eeee


Conservation:             9  9 9 9
XylA               459  EQGFAAVEFFTQTKTSYSWS----------------------  478
Cg0535             479  SVGTLAIERFMRPVSYQTFPAELLPDPVSEANKWAVPREIDR  520
Consensus_aa:           p.GhhAlE.Fhps.o.bo@s......................
Consensus_ss:             hhhhhhhhh eeeeeee  hh  hhhh
```

Fig. S1. Comparison of the calculated secondary structures of XylA (α-ketoglutarate semialdehyde dehydrogenase from *Caulobacter crescentus*) and KsaD (Cg0535, α-ketoglutarate semialdehyde dehydrogenase from *Corynebacterium glutamicum*) using PROMALS3D online (Pei *et al.,* 2008). The last two lines of each block depict the consensus amino acid sequence (Consensus_aa) and consensus predicted secondary structure (Consensus_ss), respectively. The amino acid sequences are colored according to the predicted secondary structure (red: alpha-helix, blue: beta-strand).

A

B

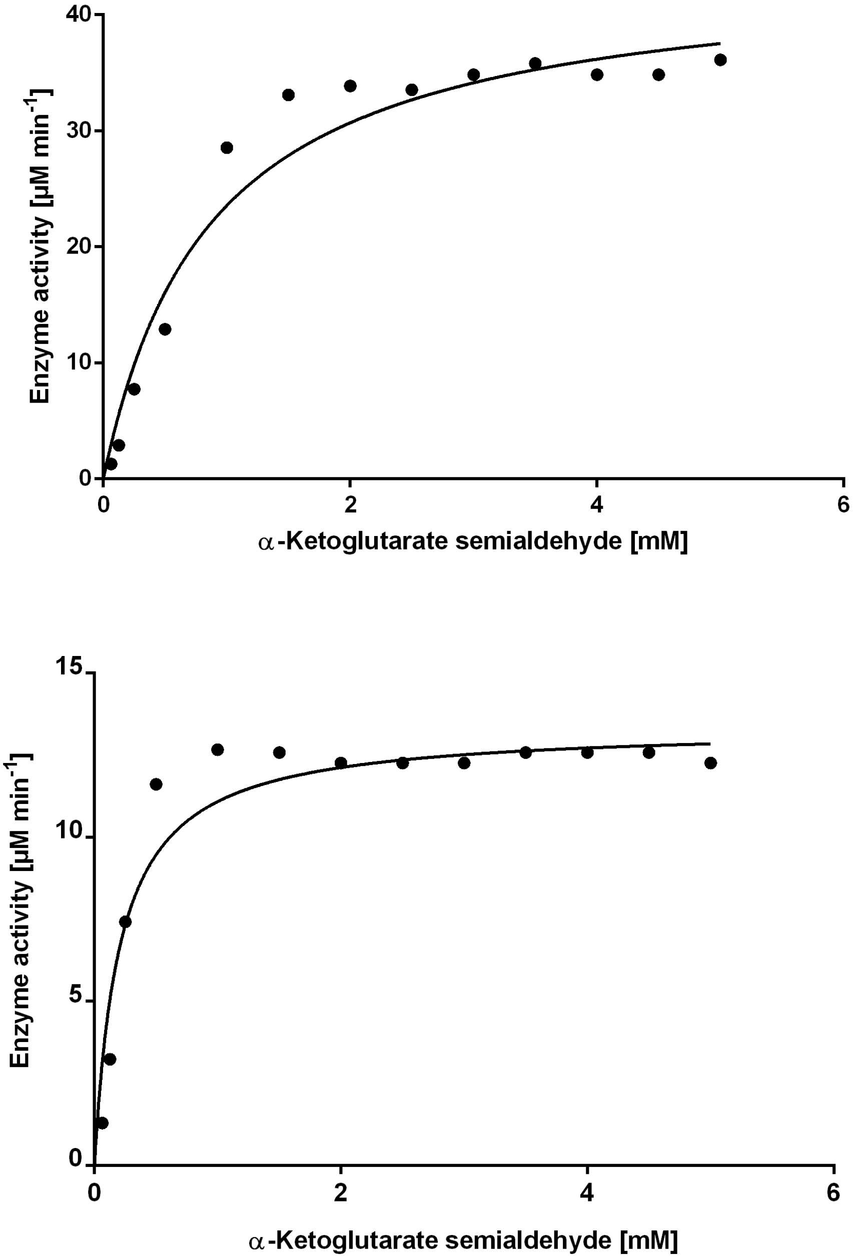

Fig. S2. Michaelis Menten plots for KsaD using NAD (A) or NADP (B) as cofactor with non-linear regression. The initial NAD(P)H generation was monitored at 340 nm and 30 °C using an Shimadzu UV-1601 Spectrophotometer. Data represent mean values from three biological replicates, respectively.

References

Pei, J., Kim, B-H., Grishin, N.V. 2008. PROMALS3D: a tool for multiple protein sequence and structure alignments. *Nucleic Acids Res.* 36 (7), 2295-2300
